# Efficient 3D AlexNet Architecture for Object Recognition Using Syntactic Patterns from Medical Images

**DOI:** 10.1155/2022/7882924

**Published:** 2022-05-20

**Authors:** Shilpa Rani, Deepika Ghai, Sandeep Kumar, MVV Prasad Kantipudi, Amal H. Alharbi, Mohammad Aman Ullah

**Affiliations:** ^1^Department of CSE, Lovely Professional University, Punjab, India; ^2^Department of CSE, Neil Gogte Institute of Technology, Hyderabad, Telangana, India; ^3^Department of Electronics and Electrical Engineering, Lovely Professional University, Punjab, India; ^4^Department of CSE, Koneru Lakshmaiah Education Foundation, Vaddeswaram, Andhra Pradesh, India; ^5^Department of E&TC, Symbiosis Institute of Technology, Symbiosis International (Deemed University), Pune, India; ^6^Department of Computer Sciences, College of Computer and Information Sciences, Princess Nourah bint Abdulrahman University, P.O. Box 84428, Riyadh 11671, Saudi Arabia; ^7^Department of Computer Science and Engineering, International Islamic University Chittagong, Chittagong, Bangladesh

## Abstract

In computer vision and medical image processing, object recognition is the primary concern today. Humans require only a few milliseconds for object recognition and visual stimulation. This led to the development of a computer-specific pattern recognition method in this study for identifying objects in medical images such as brain tumors. Initially, an adaptive median filter is used to remove the noise from MRI images. Thereafter, the contrast image enhancement technique is used to improve the quality of the image. To evaluate the wireframe model, the cellular logic array processing (CLAP)-based algorithm is then applied to images. The basic patterns of three-dimensional (3D) images are then identified from the input image by scanning the whole image. The frequency of these patterns is also used for object classification. A deep neural network is then utilized for the classification of brain tumor. In the proposed model, the syntactic pattern recognition technique is used to find the feature vector and 3D AlexNet is used for brain tumor classification. To evaluate the performance of the proposed work, three benchmark brain tumor datasets are used, i.e., Figshare, Brain MRI Kaggle, and Medical MRI datasets and BraTS 2019 dataset. The comparative analyses reveal that the proposed brain tumor classification model achieves significantly better performance than the existing models.

## 1. Introduction

Object recognition is an open-ended problem in the field of computer vision and medical image processing. For any object to recognize, a human being does not need to do much since we have multiple processing systems with billions of neurons: the brain. We require just a few milliseconds of visual stimulation to recognize an object, but the computer does not work like this because the computer needs more information for object recognition. A whole image can be given as an input but that will have computation limitations. Therefore, a generalized representation of different objects in one domain can give different identities to the same domain's objects. This generalized representation of objects can be further used for the identification of different diseases such as lung cancer and brain tumors. As image processing techniques play an essential role in the diagnosis and monitoring of patients, the biomedical field has gained much attention from researchers, and many advance artificial intelligence-based techniques are available for the early detection of the disease. However, due to the unpredictable nature of cancer, it still needs more advanced techniques.

Brain cancer is a life-threatening disease, and approximately every year, 80000 new cases are reported [1, 2]. The treatment of brain cancer always depends on the early and accurate detection of the tumor, and detection always depends on tumor type, type of pathology, and the time of the first investigation of the disease. Generally, brain tumors will irregularly propagate the brain cells [3, 4], and according to the research, three types of brain tumors are identified: glioma, meningioma, and pituitary. Most neurologists will use CAD-based models to detect and classify brain tumors [5]. According to our literature survey, most of the researchers used segmentation-based techniques to classify brain tumors [6–8]. In the last decade, statistical-based classification and detection methods are satisfactory but not a hundred percent accurate [9–13].

Therefore, in this study the image's wireframe model has been developed to detect the brain tumor from the patient's MRI. We proposed an efficient approach based on structural pattern recognition and a deep neural network because structural pattern recognition techniques can represent the patterns of simple and complex images as well. A medical image contains a lot of information, and processing these images is intricate. Hence, a wireframe model is determined and used for further processing. The wireframe model is a skeletal or edge representation of 3D objects using lines and curves [14–17]. Other basic patterns are identified in the 3D image using the syntactic pattern approach, and then, finally, these patterns and frequency of these patterns are calculated for the classification purpose. Deep learning-based algorithms play an essential role in the medical field, and according to the literature survey, the AlexNet-based architecture detects cancer in the early stage more accurately [3, 18–24]. Therefore, the 3D AlexNet architecture is used for the classification of tumor type.

This research's primary motivation was to give a new insight into the structural pattern recognition technique because structural pattern recognition techniques can deal with complex medical images [25–30]. Keeping this into mind, we targeted our few objectives, which are as follows:

- An efficient object recognition model is proposed using syntactic patterns and 3D AlexNet for medical images.
- Adaptive median filter and contrast image enhancement techniques are used to improve the quality of MRI images.
- To evaluate the wireframe model of images, the cellular logic array processing (CLAP)-based algorithm is utilized.
- Basic patterns of three-dimensional (3D) images are also identified by scanning the MRI images. The frequency of these patterns is also used for object classification.
- The performance of the proposed work is validated on three benchmark brain tumor datasets such as Figshare dataset, Brain MRI Kaggle, and Medical MRI datasets and BraTS 2019 dataset.

This study is further divided into four sections to achieve the objectives mentioned above. The second section covers the literature survey. The third and fourth sections cover the proposed methodology and results. The conclusion is discussed in the fifth section.

## 2. Literature Survey

In the processing of 3D image, superficial and volumetric features play an important role. The edge that intersects each other is referred to as volumetric edges, and the edges that separate two planes are called superficial edges. Lui et al. [6] proposed a gradient-based method for the detection of 3D edges. Rosenfeld et al. proposed an edge detection method using the surface magnitude. The construction of the wireframe model was first proposed by Mukherjee et al. [7]. Thinning and segmentation-based approach is used to evaluate the wireframe model. The adjacent segments are connected using the vertex merging method, and finally, the wireframe model is constructed.

Ren et al. [8] implemented a 3D viewpoint and shape estimation-based model to perform wireframe modeling. For the detection of the 3D viewpoints, CNN is used. Prakoonwit and Benjamin et al. [9] proposed 3D surface reconstruction and wireframe modeling by considering 2D images. In this approach, the contours of 2D images are combined and a wireframe model is created. According to the survey, most of the authors have used the viewpoint-based method for the construction of the wireframe model. Much research growth is observed in the field of statistical pattern recognition-based techniques [10], but structural-based recognition still needs researchers' attention. The structural pattern recognition technique can deal with simple and complex patterns, and these intricate patterns can be further divided into sub-patterns. Structural pattern recognition can be used for object recognition, NLP, and scene analysis [11–13].

Ali et al. [13] presented a method for the recognition of Bengali digits. Rashid and Ali [14] proposed a method in which eight directional codes are identified and these direction codes are used to convert the image into a feature vector, but the technique is only applicable for Bengali digits. Pal and Chaudhuri [15] have also proposed a method for the recognition of Bengali numerals. This technique was able to recognize the numerals without thinning and normalization operation. Jaydeb et al. [16] proposed the part of speech (POS) tagging and parsing technique method. This method is used to find the answers from the given text.

Hang [17] has presented research on the A^*∗*^ algorithm for parsing techniques and proposed an hierarchical graph method (HGM model) based on the same algorithm. This method can replace the existing virtual node method (VNM), which was used to overcome the problem of Chomsky standard forms. The performance of HGM was better than that of VNM.

For the last three decades, researchers have extensively used the machine learning approach in various areas, including medical diagnostics. There are limited numbers of studies where researchers specially targeted brain tumor diagnostic problems.  Table 1 represents the study of existing methods for brain tumor detection.

Most of the researchers used statistical pattern recognition techniques for object recognition. As per our literature survey, there is no evidence of applying the structural pattern recognition-based method in the medical field. Recently, researchers are starting using deep learning-based algorithms for the segmentation and detection of brain tumors. Hence, we used syntactic patterns for the classification of brain tumors. This is an efficient approach that helps detect and classify brain tumors in the early stage. A detailed description of the proposed methodology is discussed in the next section.

## 3. Proposed Methodology

**Figure 1:**
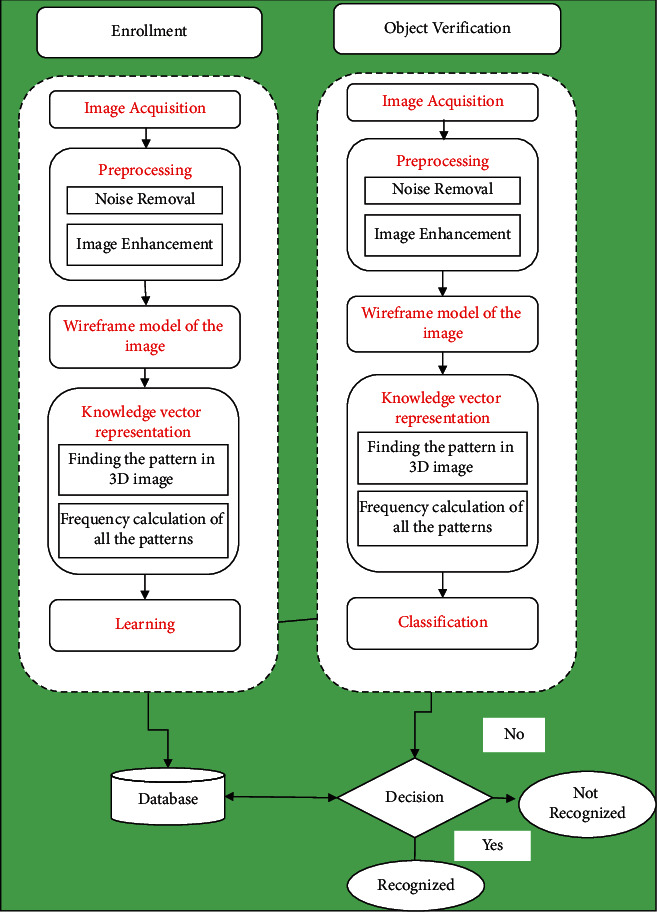
Block diagram of the proposed methodology.

This section summarizes the proposed methodology. Inspired by [2], the step-by-step procedure of the proposed classification model is designed (see Figure 1).

### 3.1. Image Preprocessing

MRI images may contain a different type of noise that can degrade the image's quality and may not provide the required and correct information to detect the cancerous tumor. An efficient denoising and image enhancement technique is required to preserve the edges and the contour of the medical images. Therefore, these techniques are directly helpful in the detection and classification of the image. In the proposed methodology, the adaptive median filter is used for image denoising purposes and an image enhancement technique is used to enhance the image.

#### 3.1.1. Denoising of the Image

Preprocessing techniques are used to remove the noise from the images. Hence, these techniques are directly helpful in the detection and classification of the image. An adaptive median filter is used to remove the noise from the image. It compares each pixel of the image to the neighborhood pixel. If any one of the pixel values is drastically different from the neighborhood pixel, then that pixel will be considered noise and the adaptive median filter will replace that noisy pixel value with the neighborhood pixel's median value. Algorithm 1 of the adaptive median filter is as follows.

#### 3.1.2. Image Enhancement

After removing the noise from the image, the next step is image enhancement. The image enhancement technique is used to improve the image's overall quality and sharpen the edges.

**Figure 2:**
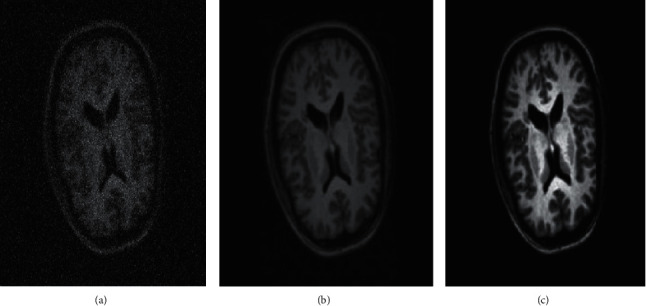
(a) Original image. (b) Denoising image. (c) Contrast-enhanced image.

In the proposed method, the contrast stretching image enhancement technique is used and is shown in Algorithm 2. This method stretches the range of intensity values, which enhances the image's quality as shown in Figure 2. First, the limit is determined over which intensity value will be extended. These limits vary between 0 and 255 in the 8 bit grayscale image. Further, a histogram of the original image is examined to determine the limits of the original image. If the input image is already covering the full possible set of values, contrast stretching is not required. Otherwise, if input image data are within the restricted range, then starching will be applied. For each image, the original value of *r* is mapped with the output value *O* using the following function.

(1) $$\begin{array}{c} O=\left(r-c\right)\left(\frac{b-a}{d-c}\right)+a. \end{array}$$

### 3.2. Wireframe of the Image

**Figure 3:**
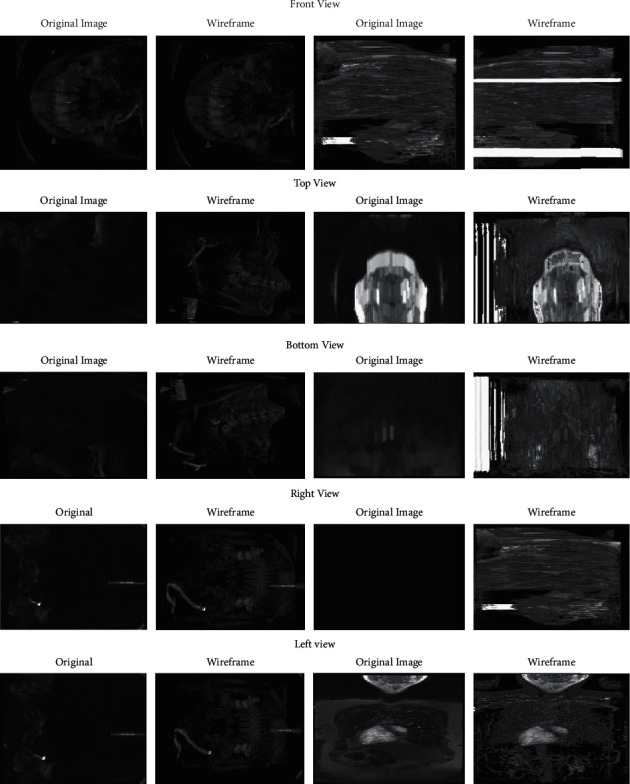
Wireframe outputs of medical images.

In this step, the preprocessed image is given as an input and then a wireframe model of the image is constructed using the CLAP-based model for 3D images [39–41]. The proposed approach works on the abrupt change in the gray level value of the neighborhood pixels. The algorithm will locate the points over which the gray level is not changing, and it works on all the planes of the 3D image. For the scanning of the 3D image, 3 × 3 × 3 size of the sliding window [42, 43] is used. It is used to verify that some pixels are establishing convex polyhedrons with every sliding window's movement. Thus, at least one pixel in every column and row provides convex polyhedrons. If such type of convex polyhedrons exists, then the central pixel will be updated by zero and the same would be repeated until it does not scan the whole 3D image using a sliding window. The performance of the algorithm is evaluated on various MRI datasets, and it is represented in Figure 3. The step-by-step flow of wireframe model is presented in Algorithm 2.

### 3.3. Knowledge Vector Representation of the Image

There are different types of shape and texture features are available in the image. The knowledge vector representation of the basic 3D patterns [44, 45] gives enough information to classify an object. In this step, we will find the basic pattern of the 3D image, and these 3D patterns will give enough information to classify an object. The following methodology is taken into consideration to identify the pattern in the image syntactic pattern recognition technique.

#### 3.3.1. Finding the Basic Patterns in a 3 × 3 × 3 Space

**Figure 4:**
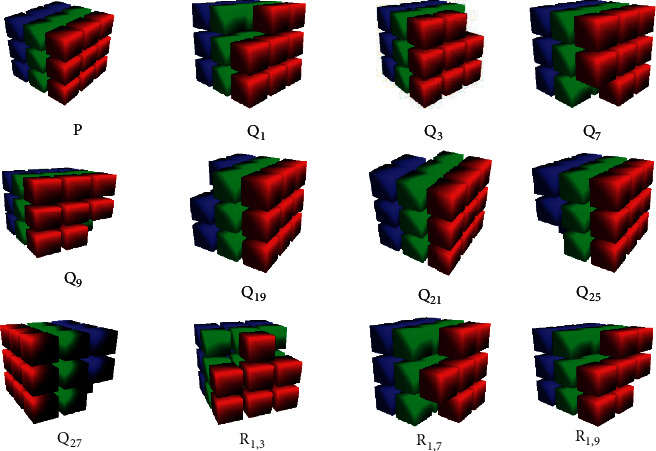
Sample images of basic patterns present in the 3D image.

In a 3D image, a central pixel is surrounded by 26 neighborhood pixels. Hence, the basic patterns have been found by combinations of 26 pixels at a time in a 3 × 3 × 3 space [46, 47]. According to our research, if we consider only the corner pixels of the image then there are 256 possible ways in which a pixel could be present in the 3 × 3 × 3 window. Sample images of the basic 3D patterns are shown in Figure 4. To differentiate these 256 possible combinations/directions, naming conventions are used and the same naming conventions are shown in Figure 4. Here, *P*_1_ represents the first pattern, which could be present in the image, and it shows that all the corner pixels are present in the image. *Q*_1_ represents that pixel 1 is not present in the 3 × 3 × 3 size of the window. In the same way, *R*_1,3_ represents that corner pixels 1 and 3 are not present in the 3 × 3 × 3 size of the window. *R*_1,7_ represents that corner pixels 3 and 7 are not present in the 3 × 3 × 3 size of the window. We need to find these patterns in the image and then calculate the frequency of the image.

**Figure 5:**
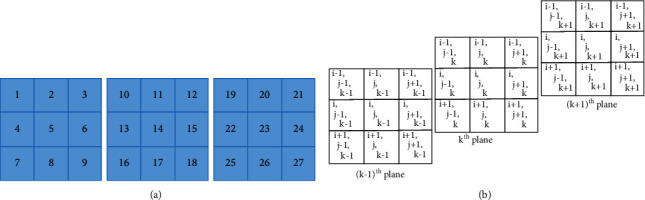
(a) 27-neighborhood structure of 3 × 3 × 3 window. (b) Representation of co-ordinates.

The naming convention is defined for all 256 patterns.  Figure 5 shows the naming convention of the particular pixel position. Figures 5(a) and 5(b) represent 27-neighborhood structure of 3 × 3 × 3 window and co-ordinates, respectively. If we consider a 3 × 3 × 3 size of an image, then a center pixel (14) can be surrounded by 26 pixels, but in our research, we considered only corner pixels, which are supposed (1, 3, 7, 9, 19, 21, 25, and 27). Hence, the possible way in which a corner pixel could be present is as follows:

(2) ∑k=08C8K=C80+C81+C82+C83+C84+C85+C86+C87+C88=256.

The possible way in which a corner pixel could be present in the image is shown in Table 2.

#### 3.3.2. Run a 3 × 3 × 3 Window on an Input Image and Check for the Patterns

The 2D slices can be combined, and it can form a 3D structure/3D volumetric data. The proposed feature extraction algorithm is written in such a way that it can work with 2D slices and 3D volumetric datasets. The proposed method takes input as a 3D volume or a sequence of 2D frames (e.g., slices in a CT scan). If it is a 2D slice and the scanning window size is 3 × 3, then a central pixel could be surrounded by eight neighborhood pixels. The naming conventions of the preferred direction are as follows: Right(R),DownRight(DR),Down(D),DownLeft(DL),Left(L),Upleft(UL),Up(U), and UpRight(UR) If we consider the sequence of 2D slices that can form a 3D structure and again the sliding window size is 3 × 3 × 3, then the central pixel could be surrounded by 26 neighborhood pixels. The naming conventions of the preferred direction are as follows:

(3) $$\begin{array}{c} \left\{\text{R,DR,D,DL,L,UL,U,UR,B,BR,BDR,BD,BDL,BL,BUL,BU,BURF,FR,FDR,FD,FDL,FL,FUL,FU,FUR}\right\}. \end{array}$$

Here, *F* represents front and *B* represents backward direction. Other conventions are the same as 2D.

The wireframe model of the image is considered as an input for this step, and the detailed explanation is given as follows.

*(1) Finding the Patterns in the Image and Calculating their Frequency Using a Stride of*3. Consider a whole image and a 3 × 3 × 3 window. This window slides over the image and finds out the pattern in which this 3 × 3 × 3 window encapsulates. If any portion of the image consists of the patterns, then the frequency value of that particular pattern would be updated. To go to the next 3×3×3 section of the image, the window moves with a stride of three. This procedure is repeated until the end of the image has been reached. The three-step stride may necessitate padding around the edges of an image in order to retrieve its information. It is to be noted that the stride of 1 is in X, *Y,* and *Z* directions. When one 3 × 3 × 3 row of an image is slid on, the next 3 × 3 × 3 row is obtained by sliding the window 3 pixels in the downward direction.

*(2) Finding the Frequency of All the Patterns Obtained (3D Pattern Frequency Vector)*. The patterns calculated in the previous step will be counted using the vocabulary of 256 patterns, and their frequency is calculated. The frequencies of all the patterns will be represented as an array. This array will be called 3D pattern frequency vector or 3D-PFV in short. The length of this vector will be 256, which corresponds to the frequency of the patterns. The frequency of the patterns written in the array should be the same and consistent. If pattern 2 is on index 1 of the 3D-PFV for one image, then it should be on the same index for all the images. The same goes for all the patterns' frequencies. This 3D-PFV representation of the image will be a mapping between the vector and the object.

3D AlexNet input layer will be a column stacked 3D picture feature vector (3D-PFV). The hidden layer will have *n* neurons with *n* weights, and the output layer will have *m* neurons for  *m* classes of objects.

#### 3.3.3. Object Recognition Using 3D Pattern Frequency Vector

**Figure 6:**
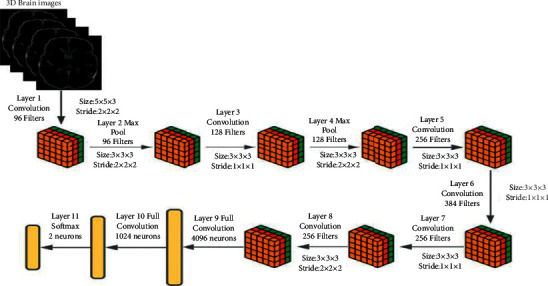
3D AlexNet architecture.

The three-dimensional knowledge vector is a vector of 256 values that saves an image's overall syntactic pattern. This image can be two- or three-dimensional. The purpose of such a vector is to get a succinct representation of a 2D or 3D image. These vectors, therefore, hold essential information regarding the shape and morphology. Therefore, theoretically, these vectors hold information about an image and thus can be used to represent it. The corresponding vector is generated using the research shown in the previous section. For each syntactic pattern, the image is calculated and fed as an integer to the 3D AlexNet. Since there are 256 patterns, the 256-dimensional vector is created, tentatively called the X-dimensional pattern frequency vector (XDPFV), where X is 3. In the proposed methodology, 3D AlexNet is used for classification purposes. AlexNet is implemented in 2012 by Alex Krizhevsky. 3D AlexNet architecture consists of 3D filters at the convolution layer and pooling layer instead of 2D filters. In the 3D AlexNet, eight layers are present, of which 5 are convolution layers and 3 are fully connected layers. 1st convolution layer is followed by the first max-pooling layer and the same for the second convolution layer. 3rd layer is connected to consecutive positions, and then, the 5th convolution layer is connected to the 3D max-pooling layer. The output generated by the 3rd max-pooling layer will become the input to the next two fully connected layers. The third fully connected layer will now be input for the soft-max classifier, and the soft-max layer consists of three class labels. The architecture of 3D AlexNet is represented in Figure 6.

## 4. Results and Discussion

In this research, we used four different brain MRI datasets. The first dataset is “Figshare brain tumor dataset,” which is publicly available [33]. This dataset consists of a total of 3064 images of 233 different patients. The second dataset is again “Brain MRI dataset,” which is publicly available on Kaggle [35]. This dataset consists of a total of 253 MRI images. The third dataset is the Medical MRI dataset, and it is collected from the Pentagram Research Institute, Hyderabad. The dataset contains 2D slices of MRI, which can be combined to make a 3D view of the images. The fourth dataset is BraTS 2019 dataset [48]. This dataset consists of the images of four different contrasts. The dataset contains four folders. The folder structure is composed of fluid-attenuated inversion recovery (FLAIR), T1, T1 contrast-enhanced, and T2.

**Figure 7:**
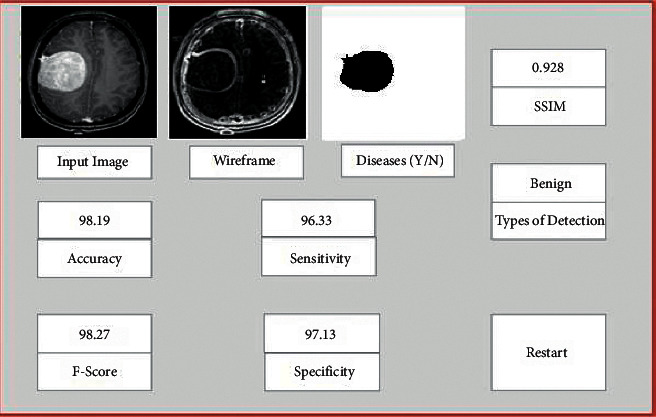
GUI of the proposed work.

To build a multi-orientation or view of each 3D model, we rotated the dataset equally in a horizontal direction and finally produced the dataset on 4*th* and 7*th* depths and multi-orientation (3, 6, 9, 12, 18, 24, and 30 rotations) occurs only on depth 7*th*. Each 3D model's multi-orientation is applied to both the training and testing datasets individually. To train the deep learning model, Keras with TensorFlow libraries has been used. The specification of the system is Windows Operating System, GPU Processor, and 32 GB RAM. We created a GUI of the proposed system for better analysis purposes, which takes MRI images as input. GUI of the proposed methodology is shown in Figure 7.

During the brain tumor classification, 3D AlexNet architecture is used, which requires values of different hyper-parameters to be set. This is required to obtain the optimum performance of the architecture. These hyper-parameters are epochs, learning rate, dropout, and batch size. The optimized values of these hyper-parameters for each problem are mentioned in Table 3. Learning rate and early stopping depend on the validation loss for every 5 to 10 epochs. In this implementation, the batch size is considered 128. The epoch is 65, and steps per each epoch are 4.

The early stopping property has been introduced in the training process, which stops the learning process at certain epochs; in case, the accuracy and loss are constant for continuous four epochs. The validation split is set to 0.1. This parameter allows the model to handle train/test data splitting on its own. 0.1  splits the data by splitting the sample dataset into 90% of training and 10% to test the samples. This allows plotting the loss and accuracy values correctly while plotting them.

The dropout technique used to ignore a random set of neurons during the training is given as 0.20. The dropout set to 20% means one in 5 inputs is excluded from each cycle. The learning rate ranges from 0 to 1. The learning rate that is too high can cause the model to converge too swiftly to a suboptimal solution. If the learning rate is too low, it causes the model to get stuck between the training processes. Simultaneously, a learning rate that is too small can cause the process to get stuck. In the proposed method, we have taken the learning rate as 0.001 so that the network learns slowly to perform object recognition on divergent input images.

### 4.1. Evaluation Parameters

The proposed methodology's performance is evaluated by the computation of accuracy, sensitivity, specificity, and F1 score defined in Table 4 [47, 49]. If accuracy, sensitivity, and specificity have a higher value, it represents the algorithm's better performance.

### 4.2. Result Analysis

MRI images were selected to evaluate the performance of the proposed system. First, a comparative analysis of the proposed wireframe model with various classical edge detection algorithms has been done, and for the comparison purpose, the Sobel, Robert, and Prewitt algorithms are taken into consideration. According to the result analysis, it is observed that the Robert and Sobel edge detection method could not produce the closed contour, leading to false tumor detection. The proposed wireframe model can generate a closed structure of the tumor and boundary of the tumor is sharp and visibility is more.

#### 4.2.1. Qualitative Analysis

Table 5 shows the subjective quality evaluation among various methods. As shown in Table 5, the proposed wireframe model is able to find the contour/wireframe model for the small object also, which is not possible in the case of Robert, Sobel, and Prewitt. As per the analysis, the proposed CLAP-based wireframe model performs better than existing methods.

#### 4.2.2. Quantitative Analysis

**Figure 8:**
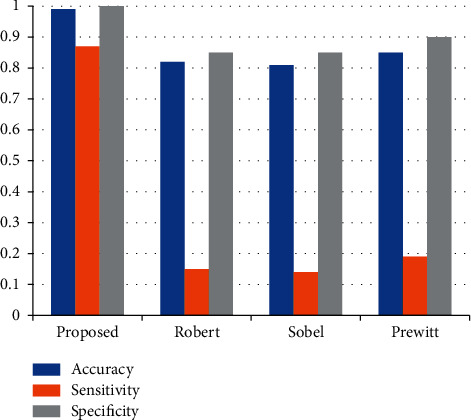
Comparative analysis of various edge detection methods with the proposed wireframe method.

**Figure 9:**
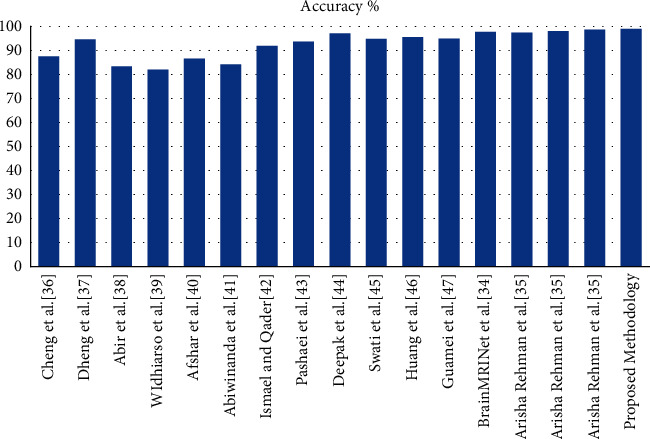
Comparative analysis of network parameters of existing systems and proposed system on Figshare dataset.

**Figure 10:**
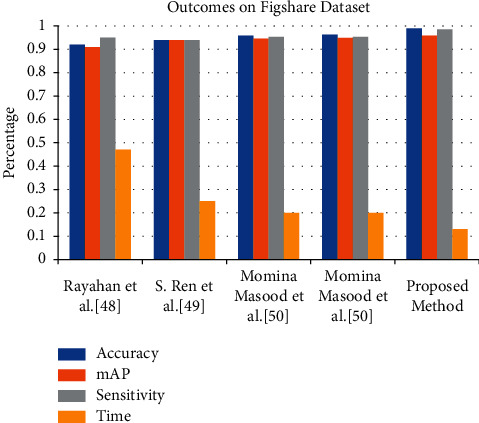
Comparative analysis of the proposed method with RCNN-based approach on the Figshare dataset.

The results obtained from the proposed wireframe model are compared with the existing edge detection method on the test images shown in Table 6. The performance measures are accuracy, sensitivity, and specificity. The accuracy of the proposed wireframe model is 99%, the sensitivity is 87%, and the specificity of 1 shows the proposed wireframe model's effectiveness for detecting the edges from the given images. The detailed statistics measures are shown in Figures 8–10.

### 4.3. Result of Classification on Different Datasets

This section represents the comparative analysis of the classification results of the proposed method with the existing methods over the same datasets. For the classification of different types of tumors, different hyper-parameters were used for the training of the proposed model. The obtained results are evaluated using different quantitative measures, which are already mentioned in Table 6.

The performance of the proposed method is evaluated on the Figshare dataset [33], Brain MRI Kaggle dataset [35], and Medical MRI dataset and BraTS 2019 dataset. The evaluation parameters are accuracy, sensitivity, specificity, precision, recall, F1 score, and mean average precision (mAP).  Table 7 shows the comparative analysis of the classification results obtained by existing methods on the Figshare dataset [33]. The highest accuracy is obtained using the fine-tune VGG-16 [61] model. The authors have used fine-tuning transfer learning, which is used to improve the efficiency of the architecture.  Figure 9 shows the graphical representation of the proposed and existing method results on the Figshare dataset.

Some authors have used the RCNN-based method for the classification of the brain tumor, and Table 8 shows the comparative analysis of the existing method on the Figshare dataset. The evaluation parameters are accuracy, mAP, sensitivity, and time. As per the comparative study, the proposed methods give better results in terms of accuracy and time on the Figshare dataset.  Figure 10 shows the graphical representation of the proposed and existing method results on the Figshare dataset.

For a better evaluation of the proposed method, we performed the experiments on two other datasets. The accuracy of the proposed method on the brain MRI dataset is 99.17%. As per the comparative study, mask RCNN performs better with an accuracy of 98.34% , but our proposed system has 1.14% higher accuracy rate than the existing system. Some other evaluation parameters are used for the validation of the proposed work. The result of the comparative analysis is shown in Table 9. The class-wise accuracy of the Figshare dataset is 98.94%, 99.18%, and 99.02% on glioma, meningioma, and pituitary classes. The accuracy of the proposed method is also evaluated on the Medical MRI dataset, and it is 99.23%. The results of the comparative analysis are shown in Table 10.

The performance of the proposed work was evaluated on the BraTS 2019 dataset.  Table 11 shows the average accuracy of the proposed method on the BraTS 2019 dataset. This section compares the proposed method with seven other existing methods, and all the researchers worked on two-class labels: LGG and HGG tumors. Zhugeet et al. [70] used deep CNN for the classification of tumor using T1, T2, and T2-FLAIR images and achieved the impressive accuracy of 97.1%. The proposed method used 3D AlexNet for the classification of tumor using T1, T1ce, T2, and T2-FLAIR images and achieved the accuracy of 96.91%, which is 0.19% lesser than the existing method [70].  Table 12 shows the class-wise performance of the various method using precision, specificity, recall, and F1 score. For low-grade glioma, the proposed 3D AlexNet achieved the highest precision of 0.925. This value is 0.062% higher than the existing pretrained ResNet mixed convolution method. For high-grade glioma and healthy subject class, the 3D AlexNet method achieved the highest precision of 0.959 and 0.998, which are higher than the existing ResNet mixed convolution method. In the same way, the performance of the proposed method in terms of recall for LGG and HGG classes is better than the existing one, but in the case of healthy subjects the recall value of the proposed method is 0.956, which is 0.039 less than the pretrained ResNet mixed convolution method. As with LGG, HGG, and healthy subjects, the best model is the exiting ResNet mixed convolution with the specificity of 0.931, 0.959, and 0.999. The F1 score value of the proposed method for LGG and HGG classes is better than the existing method, but in the classification of healthy subjects pretrained ResNet 3D achieved the highest F1 score value.

## 5. Conclusion and Future Work

In this research, a CLAP-based algorithm was used for the detection of the wireframe model. The algorithm took various input images, and their corresponding wireframe model was computed. The wireframe model finds the close surface of the given tumor image and also provides the 3D visual representation of the images. The qualitative and quantitative comparisons were performed on various edge detection algorithms. The accuracy of the CLAP-based wireframe model was found to be 99%, which is better than the majority of the existing models. Further, tumor detection and classification-based system were developed using a wireframe model and syntactic pattern recognition approach. Experiment analysis reveals that the proposed lightweight brain tumor classification model has achieved comparatively better performance than the existing state-of-art methods. Furthermore, the proposed system could combine with the existing cancer diagnostic tools to improve the robustness of the existing CAD-based systems.

## Figures and Tables

**Algorithm 1:**
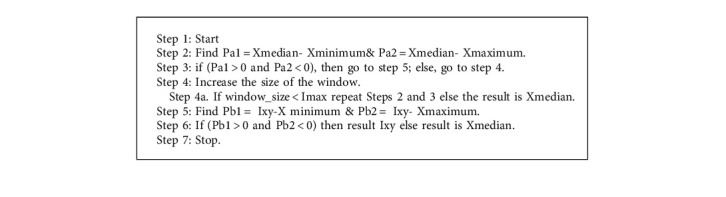


**Algorithm 2:**
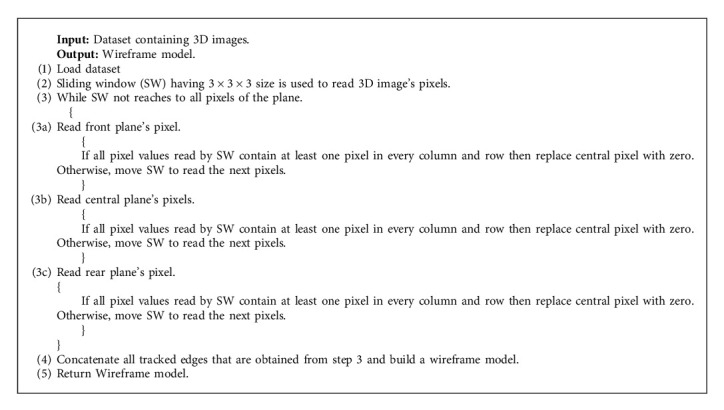
Wireframe of the image.

**Table 1:** Study of existing methodology on brain tumor detection.

S. no.	Authors and year	Methodology	Database	Remarks
1	Li et al. [18], 2019	Multi-CNN	MICCAI BraTS [31]	Accuracy = 84.3%
				Sensitivity = 82.5%
				Specificity = 97.7%

2	Addel Gawad et al. [3], 2020	Genetic algorithm	Self-dataset	FOM = 89.87%
				Accuracy = 97.71%
				Sensitivity = 94.71%
				Specificity = 98.5%

3	Mallick et al. [19], 2019	Deep wavelet auto-encoder (DWA)	Brain image dataset [32]	Accuracy = 93.14%
				Sensitivity = 94.26%
				Specificity = 92.16%
				F score = 93.15%

4	Shakeel et al. [20], 2019	AdaBoost classifier + back propagation	Self-dataset	Accuracy = 93.33%
				Sensitivity = 71.42%
				Specificity = 88.88%

5	Anitha and Murugavalli [21], 2015	K-means + DWT	Self-dataset	Accuracy = 92.43%
				Sensitivity = 95.53%
				Specificity = 50.6%

6	Mano and Anand [22], 2020	Swarm intelligence and K-means	Figshare dataset [33]	Accuracy = 98.7%
				Sensitivity = 93.4%
				Specificity = 65.1%
				F1 score = 98.7%
				JAC = 66.46%
	Swati et al. [34], 2019	VGG-19	Figshare dataset [33]	Accuracy = 94.82%

8	Khan et al. [31], 2020	VGG-16	Brain MRI dataset [35]	Accuracy = 96%
				Precision = 93%
				Recall = 100%
				F1 score = 97%
				Time = 6846sec

9	Khan et al. [31], 2020	ResNet-50	Brain MRI dataset [35]	Accuracy = 89%
				Precision = 87%
				Recall = 93%
				F1 score = 90%
				Time = 9091sec

10	Masood et al. [36], 2021	Mask-RCNN	Brain MRI dataset [35]	Accuracy = 98.34%
11	Badza and Barjaktarovic [37], 2020	CNN	Figshare dataset [33]	Accuracy = 95.40%
				F1 score = 94.94%
	Kurup et al. [38], 2019	CapsuleNet	Figshare dataset [33]	Accuracy = 92.60%
				F1 score = 93.33%

**Table 2:** Representation of a possible combination of convex polyhedrons.

Groups	Number of pixels eliminated	
Group P	Nil. Possible combination—1	P_1_ = {1,3,7,9,19,21,25,27}
Group Q	One of the corner pixels is eliminated. Possible combination—8	Q_1_ = {3,7,9,19,21,25,27},Q_3_ = {1,7,9,19,21,25,27}, Q_7_ = {1,3,9,19,21,25,27},Q_9_ = {1,3,7,19,21,25,27}, Q_19_ = {1,3,7,9,21,25,27},Q_21_ = {1,3,7,9,19,25,27}, Q_25_ = {1,3,7,9,19,21,27}, Q_27_ = {1,3,7,9,19,21,25}
Group R	Any two corner pixels are eliminated. Possible combination—28	R_1,3_ = {7,9,19,21,25,27}, R_1,7_ = {3,9,19,21,25,27}
		R_1,9_ = {3,7,19,21,25,27}, R_1,19_ = {3,7,9,21,25,27}
		R_1,21_ = {3,7,9,19,25,27}, R_1,25_ = {3,7,9,19,21,27}
		R_1,27_ = {3,7,9,19,21,25}, R_3,7_ = {1,9,19,21,25,27}
		R_3,9_ = {1,7,19,21,25,27}, R_3,19_ = {1,7,9,21,25,27}
		R_3,21_ = {1,7,9,19,25,27}, R_3,25_ = {1,7,9,19,21,27}
		R_3,27_ = {1,7,9,19,21,25}, R_7,9_ = {1,3,19,21,25,27}
		R_7,19_ = {1,3,9,21,25,27}, R_7,21_ = {1,3,9,19,25,27}
		R_7,25_ = {1,3,9,19,21,27}, R_7,27_ = {1,3,9,19,21,25}
		R_9,19_ = {1,3,7,21,25,27}, R_9,21_ = {1,3,7,19,25,27}
		R_9,25_ = {1,3,7,19,21,27}, R_9,27_ = {1,3,7,19,21,25}
		R_19,21_ = {1,3,7,9,25,27}, R_19,25_ = {1,3,7,9,21,27}
		R_19,27_ = {1,3,7,9,21,25}, R_21,25_ = {1,3,7,9,19,27}
		R_21,27_ = {1,3,7,9,19,25}, R_25,27_ = {1,3,7,9,19,21}
Group S	Any three pixels are eliminated. Possible combination—56	Same way for these groups also
Group T	Any four pixels are eliminated. Possible combination—70	
Group U	Any five pixels are eliminated. Possible combination—56	
Group V	Any six pixels are eliminated. Possible combination—28	
Group W	Any seven pixels are eliminated. Possible combination—8	
Group X	All corner pixels are eliminated. Possible combination—1	**X**_1,3,7,9,19,21,25,27_ = { }

**Table 3:** Details of 3DAlexNet classifier.

Parameter name	Values
Epochs	65
Learning rate	0.001
Dropout	0.20
Batch size	128
No. of convolution	8
No. of fully connected layer	3
Pooling layer	Max pooling
Pooling layer window size	2

**Table 4:** List of evaluation parameters and their formula.

S. No.	Parameters name	Formula
1	Accuracy	Accuracy=TP+TN/(TP+TN+FP+FN)
2	Specificity	Specificity=TN/TN+TP
3	Sensitivity	Sensitivity=TP/TP+FN
4	F1 score	F1 − score=2*∗*precision*∗*recall/precision+recall

TP = true positive, TN = true negative, FP =false positive, and FN =false negative.

**Table 5:** Comparative analysis of various edge detection methods on the different 3D views of the estimated tumor inside a cancerous brain.

Original image	Wireframe	Robert	Sobel	Prewitt
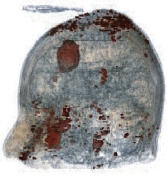	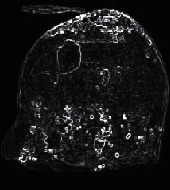	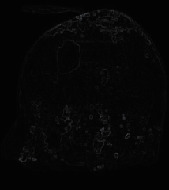	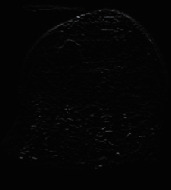	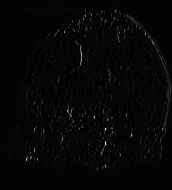
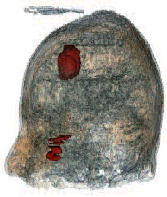	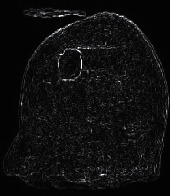	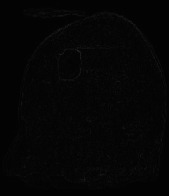	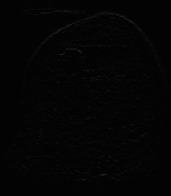	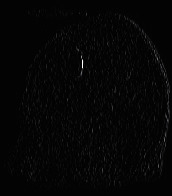
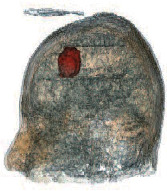	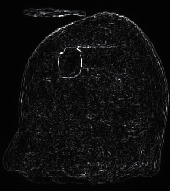	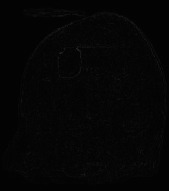	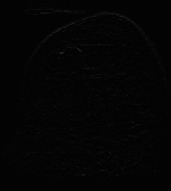	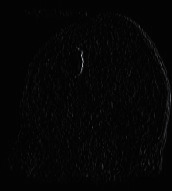
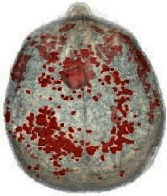	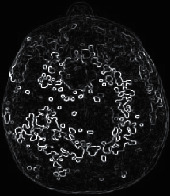	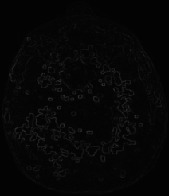	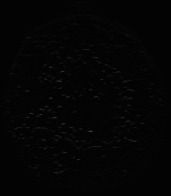	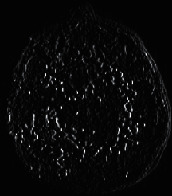
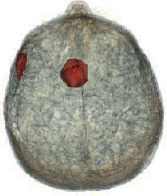	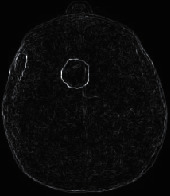	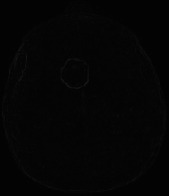	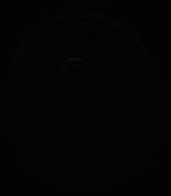	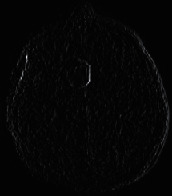

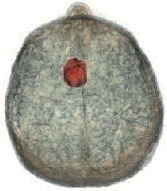	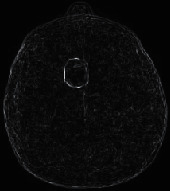	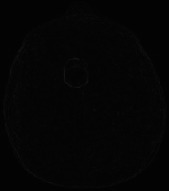	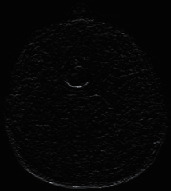	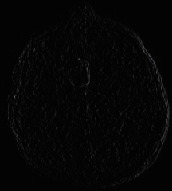

**Table 6:** Performance analysis of various edge detection methods with the proposed wireframe method.

Method	Accuracy	Sensitivity	Specificity
Proposed	0.99	0.87	1
Robert	0.82	0.15	0.85
Sobel	0.81	0.14	0.85
Prewitt	0.85	0.19	0.90

**Table 7:** Result analysis of the proposed work with other state-of-art methodology on Figshare dataset.

Method	Model	Accuracy (%)
Cheng et al. [50]	SVM	87.54
Dheng et al. [51]	SVM	94.68
Abir et al. [52]	PNN	83.33
WIdhiarso et al. [53]	CNN	82
Afshar and Plataniotis [54]	CapsNet	86.56
Abiwinanda et al. [55]	CNN	84.19
Ismael and Qader [56]	MPNN	91.90
Pashaei and Jazayeri [57]	ELM	93.68
Deepak and Ameer [58]	GoogleNet and SVM	97.10
Swati et al. [34]	VGG-19	94.82
Huang et al. [59]	CNN based on complex networks	95.49
Gumaei et al. [60]	GIST descriptor and ELM	94.93
Chakrabarty [35]	Attention module, hyper column technique, residual block	97.69
Arisha Rehman et al. [61]	Fine-tune AlexNet	97.39
Arisha Rehman et al. [61]	Fine-tune GoogleNet	98.04
Arisha Rehman et al. [61]	Fine-tune VGG-16	98.69
Proposed methodology	3D AlexNet	**99.04**

**Table 8:** Performance comparison of the proposed method with existing RCNN-based methods on Figshare dataset.

Method	Model	Evaluation metrics			
		Accuracy	mAP	Sensitivity	Time (s)
Rayahan et al. [62]	RCNN	0.920	0.910	0.950	0.47
Ren et al. [63]	Faster RCNN	0.940	0.940	0.940	0.25
Masood et al. [36]	ResNet-50	0.959	0.946	0.953	0.20
Masood et al. [36]	DenseNet-41	0.963	0.949	0.953	0.20
	Proposed method	**0.990**	0.958	0.985	0.13

**Table 9:** Result analysis of the proposed work with other state-of-art methodology on the Figshare dataset.

Method	Model	Classes	Accuracy (%)	Sensitivity (%)	Specificity (%)	F1 score (%)
Rehman et al. [61]	Freeze AlexNet	—	91.21	90.48	95.48	—
Rehman et al. [61]	Freeze GoogleNet	—	93.46	95.38	97.74	—
Rehman et al. [61]	Freeze VGG-16	—	89.76	87.81	94.64	—
Pashaei et al. [64]	CNN	—	93.68	—	—	93.00
Badza and Barjaktarovic [37]	CNN	—	95.40	—	—	94.94
Kurup et al. [38]	CapsuleNet		92.60	—	—	93.33
Srinivasan et al. [65]	GLCM and wavelet packets		93.30	—	—	72.00
Proposed method		Glioma	98.94	98.94	98.79	97.3
		Meningioma	99.18	98.11	99.01	98.7
		Pituitary	99.02	98.72	98.19	98.69

**Table 10:** Result analysis of the proposed work with other state-of-art methodology on the Brain MRI Kaggle dataset and Medical MRI dataset.

Method	Model	Accuracy	Precision	Recall	F1 score	Time (sec)
Swati et al. [34]	VGG-19	0.94	—	—	—	—
Masood et al. [36]	Mask-RCNN	0.98	—	—	—	—
Hassan et al. [31]	CNN	1.0	1.0	1.0	1.0	3085
Hassan et al. [31]	VGG-16	0.96	0.93	1.0	0.97	6846
Hassan et al. [31]	ResNet-50	0.89	0.87	0.93	0.90	9091
Hassan et al. [31]	Inception-v3	0.75	0.77	0.71	0.74	5630
Proposed work on Brain MRI dataset	**3D AlexNet**	**0.99**	0.97	0.94	0.91	3706
Proposed work on Medical MRI dataset	**3D AlexNet**	**0.99**	0.98	0.95	0.93	3891

**Table 11:** Result analysis of the proposed work on the BraTS 2019 dataset.

Study	Method	Contrast	Accuracy (%)
Shahzadi et al. [66]	CNN with LSTM	T2-FLAIR	84.00
Pei et al. [67]	CNN	T1, T1ce, T2, T2-FLAIR	74.9
Ge et al. [68]	Deep CNN	T1, T2, T2-FLAIR	90.87
Mzoughi et al. [69]	Deep CNN	T1, T1ce, T2, T2-FLAIR	96.59
Zhuge et al. [70]	Deep CNN	T1, T2, T2-FLAIR	97.1
Ouerghi et al. [71]	Random forest	T1, T2, T2-FLAIR	96.5
Chatterjee et al. [72]	Pretrained ResNet mixed convolution	T1ce	96.98
Proposed	3D AlexNet	T1, T1ce, T2, T2-FLAIR	96.91

**Table 12:** Result analysis of the proposed work with other state-of-art methodology on the BraTS 2019 dataset.

Study	Method	Classes	Precision (%)	Recall (%)	Specificity (%)	F1 score (%)
Chatterjee et al. [72]	ResNet 3D	Low-grade glioma	0.798	0.920	0.907	0.854
		High-grade glioma	0.933	0.828	0.962	0.877
		Healthy subjects	0.993	0.99	0.99	0.99

Chatterjee et al. [72]	Pretrained ResNet 3D	Low-grade glioma	0.781	0.855	0.911	0.814
		High-grade glioma	0.896	0.835	0.937	0.863
		Healthy subjects	1.00	0.999	1.00	0.999

Chatterjee et al. [72]	ResNet(2 + 1)D	Low-grade glioma	0.786	0.914	0.902	0.844
		High-grade glioma	0.930	0.822	0.962	0.873
		Healthy subjects	0.994	0.990	0.997	0.992

Chatterjee et al. [72]	Pretrained ResNet(2 + 1)D	Low-grade glioma	0.841	0.910	0.931	0.873
		High-grade glioma	0.928	0.870	0.959	0.897
		Healthy subjects	0.999	0.998	0.999	0.999

Chatterjee et al. [72]	ResNet mixed convolution	Low-grade glioma	0.747	0.886	0.855	0.773
		High-grade glioma	0.911	0.750	0.963	0.823
		Healthy subjects	0.994	0.976	0.997	0.985

Chatterjee et al. [72]	Pretrained ResNet mixed convolution	Low-grade glioma	0.863	0.931	0.912	0.894
		High-grade glioma	0.944	0.883	0.936	0.912
		Healthy subjects	0.997	0.995	0.996	0.996

Proposed	**3D AlexNet**	Low-grade glioma	0.925	0.946	0.963	0.904
		High-grade glioma	0.959	0.895	0.998	0.919
		Healthy subjects	0.998	0.956	0.978	0.968

## Data Availability

The data used to support the findings of this study are available from the corresponding author upon request.

## References

[1] Li M., Kuang L., Xu S. (2019). Brain tumor detection based on multimodal information fusion and convolutional neural network. *IEEE Access*.

[2] Rani S., Lakhwani K., Kumar S. (2021). Knowledge vector representation of three dimensional convex polyhedrons and reconstruction of medical images using knowledge vector. https://assets.researchsquare.com/files/rs-380191/v1_covered.pdf?c=1631868418.

[3] Abdel-Gawad A. H., Said L. A., Radwan A. G. (2020). Optimized edge detection technique for brain tumor detection in MR images. *IEEE Access*.

[4] Han C., Rundo L., Araki R. (2019). Combining noise-to-image and image-to-image GANs: brain MR image augmentation for tumor detection. *IEEE Access*.

[5] Wang W., Bu F., Lin Z., ShuangqingZhai (2020). Learning methods of convolutional neural network combined with image feature extraction in brain tumor detection. *IEEE Access*.

[6] Liu H. K. (1977). Two-and three-dimensional boundary detection. *Computer Graphics and Image Processing*.

[7] Mukherjee J., Das P. P., Chatterji B. N. (1990). An algorithm for the extraction of the wire frame structure of a three-dimensional object. *Pattern Recognition*.

[8] Ren X., Jiang L., Tang X., Liu W. (2019). 3D wireframe modeling and viewpoint estimation for multi-class objects combining deep neural network and deformable model matching. *Applied Sciences*.

[9] Prakoonwit S., Benjamin R. (2007). 3D surface point and wireframe reconstruction from multiview photographic images. *Image and Vision Computing*.

[10] Gonzalez R. C., Woods R. E. (2002). *Digital Image Processing*.

[11] Tanaka E. (1995). Theoretical aspects of syntactic pattern-recognition. *Pattern Recognition*.

[12] Memon N., Neuhoff D. L., Shende S. (2000). An analysis of some common scanning techniques for lossless image coding. *IEEE Transactions on Image Processing*.

[13] Moshad M. A., Ali M. M. Recognition of handwritten Bangla digits by intelligent regional search method.

[14] Rashid M., Ali M. M. On line recognition of handwritten characters using 1-dim, 1-dim DP approach.

[15] Pal U., Chaudhuri B. B., Tan T., Shi Y., Gao W. Automatic recognition of unconstrained off-line Bangla handwritten numerals.

[16] Jaydeb S., Billah M., Mamun M. A. Textual question answering for semantic parsing in natural language processing.

[17] Hang L. Q. A hierarchical graph method using in A^∗^ algorithm for Vietnamese parsing technique.

[18] Li M., Kuang L., Xu S., Sha Z. (2019). Brain tumor detection based on multimodal information fusion and convolutional neural network. *IEEE Access*.

[19] Mallick P. K., Ryu S. Ho, Kumar Satapathy S., Mishra S., Nguyen G. N., Tiwari P. (2019). Brain MRI image classification for cancer detection using deep wavelet autoencoder-based deep neural network. *IEEE Access*.

[20] Shakeel P. M., Al-Feel H., Manogaran G., Baskar S. (2019). Neural network-based brain tumor detection using wireless infrared imaging sensor. *IEEE Access*.

[21] Anitha V., Murugavalli S. J. I. C. V. (2016). Brain tumor classification using two-tier classifier with adaptive segmentation technique. *IET Computer Vision*.

[22] Mano A., Anand S. (2020). Method of multi-region tumour segmentation in brain MRI images using grid-based segmentation and weighted bee swarm optimization. *IET Image Processing*.

[23] Kumar S., Singh S., Kumar J. (2018). Live detection of face using machine learning with multi-feature method. *Wireless Personal Communications*.

[24] Kumar S., Singh S., Kumar J. (2018). Automatic live facial expression detection using genetic algorithm with haar wavelet features and SVM. *Wireless Personal Communications*.

[25] Kumar S., Singh S., Kumar J. (2021). Face spoofing detection using improved SegNet architecture with a blur estimation technique. *International Journal of Biometrics*.

[26] Raja R., Kumar S., Rani S., Ramya Laxmi K. (2020). Lung segmentation and nodule detection in 3D medical images using convolution neural network. *Artificial Intelligence and Machine Learning in 2D/3D Medical Image Processing*.

[27] Shilpa C., Lakhwani K., Agrwal S. (2012). An efficient hybrid technique of feature extraction for facial expression recognition using AdaBoost Classifier. *International Journal of Engineering Research and Technology*.

[28] Swathi A., Rani S. Intelligent fatigue detection by using ACS and by avoiding false alarms of fatigue detection.

[29] Jain A., Kumar A., Sharma S. (2015). Comparative design and analysis of mesh, torus and ring NoC. *Procedia Computer Science*.

[30] Ghai D., Kumar Gianey H., Jain A., Singh Uppal R. (2020). Quantum and dual-tree complex wavelet transform-based image watermarking. *International Journal of Modern Physics B*.

[31] Khan H. A., Wu J., Mushtaq M., Mushtaq M. U. (2020). Brain tumor classification in MRI image using convolutional neural network[J]. *Mathematical Biosciences and Engineering*.

[32] Menze B. H., Jakab A., Bauer S. (2015). The multimodal brain tumor image segmentation benchmark (BRATS). *IEEE Transactions on Medical Imaging*.

[33] Cheng J. (2020). Brain tumor dataset. https://figshare.com/articles/brain_tumor_dataset/1512427.

[34] Swati Z. N. K., Zhao Q., Kabir M. (2019). Brain tumor classification for MR images using transfer learning and fine-tuning. *Computerized Medical Imaging and Graphics*.

[35] Chakrabarty N. (2019). Brain MRI images dataset for brain tumor detection. https://www.kaggle.com/navoneel/brain-mri-images-forbrain-tumor-detection.

[36] Masood M., Nazir T., Nawaz M. (2021). A novel deep learning method for recognition and classification of brain tumors from MRI images. *Diagnostics*.

[37] Badža M. M., Barjaktarović M. Č. (2020). Classification of brain tumors from MRI images using a convolutional neural network. *Applied Sciences*.

[38] Kurup R. V., Sowmya V., Soman K. P. Effect of data pre-processing on brain tumor classification using capsulenet.

[39] Shilpa R., Lakhwani K., Kumar S. Three-dimensional wireframe model of medical and complex images using cellular logic array processing techniques.

[40] Kaur M., Singh D. (2021). Multiobjective evolutionary optimization techniques based hyperchaotic map and their applications in image encryption. *Multidimensional Systems and Signal Processing*.

[41] Bilandi N., Verma H. K., Dhir R. (2020). AHP–neutrosophic decision model for selection of relay node in wireless body area network. *CAAI Transactions on Intelligence Technology*.

[42] Xu Y., Li Y., Li C. (2021). Electric window regulator based on intelligent control. *Journal of Artificial Intelligence and Technology*.

[43] Jiang J., Hu L. (2020). Decentralised federated learning with adaptive partial gradient aggregation. *CAAI Transactions on Intelligence Technology*.

[44] Kaur M., Singh D. (2021). Multi-modality medical image fusion technique using multi-objective differential evolution based deep neural networks. *Journal of Ambient Intelligence and Humanized Computing*.

[45] Mondal S. C., Marquez P. L. C., Osman Tokhi M. (2021). Analysis of mechanical adhesion climbing robot design for wind tower inspection. *Journal of Artificial Intelligence and Technology*.

[46] Kaushik H., Singh D., Kaur M., Alshazly H., Zaguia A., Hamam H. (2021). Diabetic retinopathy diagnosis from fundus images using stacked generalization of deep models. *IEEE Access*.

[47] Aljunid, Fadhel M., Huchaiah M. D. (2020). Multi-model deep learning approach for collaborative filtering recommendation system. *CAAI Transactions on Intelligence Technology*.

[48] Available online (2019). Available online. https://https://www.kaggle.com/aryashah2k/brain-tumor-segmentation-brats-2019.

[49] Singh P. K. (2022). Data with non-Euclidean geometry and its characterization. *Journal of Artificial Intelligence and Technology*.

[50] Cheng J., Huang W., Shuangliang Cao R. (2015). Enhanced performance of brain tumor classification via tumor region augmentation and partition. *PLoS One*.

[51] Deng J., Dong W., Socher R., Li L.-J., Li K., Fei-Fei L. ImageNet: a large-scale hierarchical image database.

[52] Abir T. A., Siraji J. A., Ahmed E., Khulna B. (2018). Analysis of a novelMRI based brain tumour classification using probabilistic neural network. *Int. J. Sci. Res. Sci. Eng. Technology*.

[53] Widhiarso W., Yohannes Y., Prakarsah C. (2018). Brain tumor classification using gray level co-occurrence matrix and convolutional neural network. *Indones. J. Electron. Instrum. Syst*.

[54] Afshar A. M., Plataniotis K. N. Brain tumor type classification via capsule networks.

[55] Abiwinanda N., Hanif M., Hesaputra S. T., Handayani A., Mengko T. R. Brain tumor classification using convolutional neural network.

[56] Ismael M. R., Abdel-Qader I. Brain tumor classification via statistical features and back-propagation neural network.

[57] Pashaei H. S., Jazayeri N. Brain tumor classification via convolutional neural network and extreme learning machines.

[58] Deepak S., Ameer P. (2019). Brain tumor classification using deep CNN features via transfer learning. *Computers in Biology and Medicine*.

[59] Huang Z., Du X., Chen L. (2020). Convolutional neural network based on complex networks for brain tumor image classification with a modified activation function. *IEEE Access*.

[60] Gumaei A., Hassan M. M., Hassan M. R., Alelaiwi A., Fortino G. (2019). A hybrid feature extraction method with regularized extreme learning machine for brain tumor classification. *IEEE Access*.

[61] Rehman A., Naz S., Imran Razzak M., Akram F., Imran M. (2020). A deep learning-based framework for automatic brain tumors classification using transfer learning. *Circuits, Systems, and Signal Processing*.

[62] Rayhan F., Fr-MRInet (2018). A deep convolutional encoder-decoder for brain tumor segmentation with relu-RGB and slidingwindow. *International Journal of Computer Application*.

[63] Ren S., He K., Girshick R. B., Sun J. (2017). Faster R-CNN: towards real-time object detection with region proposal networks. *IEEE Transactions on Pattern Analysis and Machine Intelligence*.

[64] Pashaei A., Sajedi H., Jazayeri N. Brain tumor classification via convolutional neural network and extreme learning machines.

[65] Srinivasan K., Nandhitha N. M. (2019). Development of deep learning algorithms for brain tumor classification using GLCM and wavelet packets. *Caribbean Journal of Science*.

[66] Shahzadi I., Tang T. B., Meriadeau F., Quyyum A. Cnn-lstm: cascaded framework for brain tumour classification.

[67] Pei L., Vidyaratne L., Hsu W. W., Rahman M. M., Iftekharuddin K. M. Brain tumor classification using 3d convolutional neural network.

[68] Ge C., Gu Y. H., Jakola A. S., Yang J. Deep learning and multi-sensor fusion for glioma classification using multistream 2d convolutional networks.

[69] Mzoughi H., Njeh I., Wali A., Slima M. B., BenHamida A. (2020). Deep multi-scale 3d convolutional neural network (cnn) for mri gliomas brain tumor classification. *Journal of Digital Imaging*.

[70] Zhuge Y., Ning H., Mathen P., Cheng J. Y., Krauze A. V. (2020). Automated glioma grading on conventional mri images using deep convolutional neural networks. *Medical Physics*.

[71] Ouerghi H., hak Lee S., Kim J. (2021). Glioma classification via mr images radiomics analysis. *The Visual Computer*.

[72] Chatterjee S., Nizamani F. A., Nürnberger A., Speck O. (2022). Classification of brain tumours in MR images using deep spatiospatial models. *Scientific Reports*.

